# Apolipoprotein E4 Is Deficient in Inducing Macrophage ABCA1 Expression and Stimulating the Sp1 Signaling Pathway

**DOI:** 10.1371/journal.pone.0044430

**Published:** 2012-09-11

**Authors:** Emmanuel Ugochukwu Okoro, Yanfeng Zhao, ZhongMao Guo, Lichun Zhou, Xinghua Lin, Hong Yang

**Affiliations:** Department of Physiology, Meharry Medical College, Nashville, Tennessee, United States of America; University of Padova, Italy

## Abstract

ATP binding cassette A1 (ABCA1) is a membrane protein that promotes cellular cholesterol efflux. Using RAW 264.7 macrophages, we studied the relative effects of apolipoprotein (apo) E3 and apoE4 on ABCA1 and on the signaling pathway that regulates its expression. Both lipid-associated and lipid-free apoE4 forms induced ∼30% lower levels of ABCA1 protein and mRNA than apoE3 forms. Phosphorylated levels of phosphoinositol 3-kinase (PI3K), protein kinase Cζ (PKCζ) and specificity protein 1 (Sp1) were also lower when treated with apoE4 compared to apoE3. The reduced ability of apoE4 to induce ABCA1 expression, PKCζ and Sp1 phosphorylation were confirmed in human THP-1 monocytes/macrophages. Sequential phosphorylation of PI3K, PKCζ and Sp1 has been suggested as a mechanism for upregulation of ABCA1 expression. Both apoE3 and apoE4 reduced total cholesterol and cholesterol esters in lipid-laden RAW 264.7 cells, and induced apoAI-mediated cholesterol efflux. However, the cholesterol esters and cholesterol efflux in apoE4-treated cells were ∼50% and ∼24% lower, respectively, compared to apoE3-treated cells. Accumulation of cholesterol esters in macrophages is a mechanism for foam cell formation. Thus the reduced ability of apoE4 to activate the PI3K-PKCζ-Sp1 signaling pathway and induce ABCA1 expression likely impairs cholesterol ester removal, and increases foam cell formation.

## Introduction

Apolipoprotein E (apoE), also known as arginine-rich apoprotein, was first characterized as a protein moiety of very low-density lipoproteins (VLDL) [1], [2]. Further studies have shown that it is a constituent of chylomicron remnants [3] and high density lipoproteins (HDL) [3]–[6], in addition to being present in a lipid-free state [7]. Originally, isoelectric focusing of the isolated proteins [8] demonstrated that human apoE contains more than one form; isoforms were subsequently named from the most acidic to the least acidic, *i.e.,* E1 is more acidic than E2, while E2 is more acidic than E3, and so on [9]–[11]. ApoE3, with an allelic frequency of about 68–87%, depending on the population under study [12], [13], is considered the normal isoform. The two other common isoforms, apoE2 and apoE4, occur at about a 7–10% and 10–22% allelic frequency [12], [13], respectively, and are found in higher frequencies in people of African descent. Remarkably, apoE3 differs from the minor isoforms, E2 and E4, by a single amino acid. Specifically, the Arg^158^ in apoE3 is mutated to Cys^158^ in apoE2, while the Cys^112^ in apoE3 is mutated to Arg^112^ in apoE4 [14], [15].

Lipid-associated, but not lipid-free apoE, binds low-density lipoprotein receptor (LDLR) [16]–[18] as well as several other LDLR-related proteins (LRPs) [16], [19]–[21]. By binding to these receptors, apoE initiates receptor-mediated endocytosis of VLDL [21] and chylomicron remnants [22]. The internalized lipoproteins and receptors disintegrate in early sorting endosomes. Once there, the lipid core and apoB components are further directed to lysosomes for degradation, while apoE is efficiently recycled back to the extracellular space [23], often in association with a high-density lipoprotein (HDL) particle [24], [25]. Recycling of apoE with HDL in liver cells can supply chylomicron remnants with apoE in the postprandial state. In addition, apoE recycling in cells is accompanied with cholesterol efflux [26]. Impaired recycling of apoE in macrophages thus might result in intracellular cholesterol accumulation and trigger foam cell formation [27].

As compared to apoE3 protein, apoE4 appears to promote greater binding and uptake of lipoproteins by hepatocytes [28]. Also the plasma clearance of apoB-carrying lipoproteins is greater when apoE4 is present compared to apoE3 [29]. However, apoE4 is defective in the recycling process. Individuals carrying the apoE4 allele manifest elevated postprandial plasma lipids [30] and have a higher susceptibility to coronary heart diseases compared to those carrying apoE3 [31]–[33]. This deficiency in recycling may contribute to postprandial hyperlipidemia and the pro-atherogenic effect of apoE4 [34].

Another function of apoE in lipid metabolism is to promote excess cholesterol and phospholipid efflux from cells. There is evidence that lipid-associated apoE3 promotes greater cholesterol efflux than lipid-associated apoE4 in hepatocytes [27], [35]. Similarly, lipid-free apoE3 promotes greater cholesterol efflux than apoE4 from neurons and astrocytes [36], [37]. Various mechanisms have been described to explain the involvement of apoE in cholesterol efflux. For example, many cells, including macrophages, secrete apoE in response to lipid-loading [38]–[41]. The apoE, through autocrine and/or paracrine process(es), accepts cholesterol released from macrophages, resulting in assembly of nascent HDL [24], [40]. In addition, up-regulation of ATP binding cassette A1 (ABCA1) expression has been suggested as another mechanism whereby apoE induces cholesterol efflux. ABCA1 is a plasma membrane protein that exports excess cholesterol and phospholipids [35], [42], [43]. Recent studies from our laboratory demonstrated that mouse apoE/apoB-carrying lipoproteins and lipid-free human apoE3 increased ABCA1 mRNA and protein levels in mouse macrophages [44]–[46], while apoE-free and apoB-carrying lipoproteins were less effective at inducing ABCA1 expression [44]. Our previous study also demonstrated that sequential activation of phosphatidylinositol 3-kinase (PI3K), protein kinase Cζ (PKCζ) and specificity protein 1 (Sp1) was at least partially responsible for the increased ABCA1 expression induced by apoE/apoB-carrying lipoproteins [45] and lipid-free apoE3 [46].

In the present report, we compared the effect of apoE3 and apoE4 on ABCA1 expression and the PI3K-PKCζ-Sp1 signaling cascade. Our data demonstrated that apoE4 is less effective at inducing ABCA1 expression and activating the PI3K-PKCζ-Sp1 signaling pathway, as compared to apoE3. In addition, apoE4 is comparatively inefficient at removing cholesterol from lipid-laden macrophages. A deficiency in ABCA1 expression and consequent reduction in cholesterol efflux might contribute to the increased prevalence of atherosclerotic cardiovascular diseases in carriers of apoE4.

## Materials and Methods

### Ethics Statement

All procedures for handling animals were conducted following protocols approved by the Institutional Animal Care and Use Committee at Meharry Medical College, protocol number 10020IE0018, entitled “Human ApoE4 and Foam Cell Formation.”

### Chemicals and Reagents

ApoE3 and apoE4 proteins were purchased from Leinco Technologies (St. Louis, Missouri); the lyophilized powders were dissolved to 1mg/ml in 5 mM phosphate buffered saline (PBS; pH 7.8) using 0.5 mM dithiothreitol to prevent disulfide bridges. These proteins were generated using recombinant technology and purified from *E. Coli*. The purity of both apoE3 nd apoE4 proteins is >90% (Leinco Technologies). The amount of the proteins was verified by western blotting and a BCA assay kit (Thermo Scientific, Rockford, IL). Antibodies against ABCA1 (sc-58219-M), Sp1 (sc-14027-R), and phosphorylated PKCζ (sc-12894-R) were purchased from Santa Cruz Biotechnologies (Santa Cruz, CA). Phosphorylated PI3K antibody (#14228S) was purchased from Cell Signaling (Boston, MA). Mouse-derived RAW 264.7 and human-derived THP-1 macrophage cell lines were obtained from the American Type Culture Collection (ATCC) (Manassas, VA). Pierce BCA Protein Assay Kit was obtained from Thermo Scientific (Rockford, IL). Protease inhibitor cocktail was purchased from Roche Applied Science (Indianapolis, IN). Dulbecco’s modified Eagle’s medium (DMEM), fetal bovine serum (FBS), Trizol reagent, and penicillin/streptomycin, as well as the primers for amplification of ABCA1 and 3-phosphate dehydrogenase (GAPDH) were purchased from Invitrogen (Carlsbad, CA). A High Capacity cDNA Reverse Transcription kit was purchased from Applied Biosystems (Carlsbad, CA). Cholesterol assay kits were purchased from Wako Chemicals (Richmond, VA). Heparin was purchased from Sigma-Aldrich (St Louis, MO), while [1,2-^3^H(N)]-cholesterol was obtained from Perkin Elmer (Waltham, MA).

### Mouse Plasma Lipoprotein Isolation

ApoE knockout (*apoE^−/−^)* mice (3–4 months old) were obtained from The Jackson Laboratory (Bar Harbor, ME) and fed a chow diet containing approximately 5% fat and 19% protein by weight (Harlan Teklad, Madison, WI). Approximately 0.5 ml of blood was collected from the posterior vena cava of mice under anesthesia with ketamine hydrochloride (100 mg/ml) at 0.8 µl/g body weight. To minimize oxidation and clotting, collected blood was immediately mixed with 50 µM butylated hydroxytoluene and 2 mM EDTA, and cooled on ice. Mouse plasma was overlaid with a potassium bromide (KBr) gradient solution (*d*: 1.063) and centrifuged at 120,000 rpm (511,258 g av.) for 2 h. Lipoproteins with density <1.063 g/ml were collected, dialysed in PBS (pH 7.4) containing 10 mM EDTA for 48 h at 4°C and filtered through a 0.45-µm filter [45], [47]. These lipoproteins include VLDL, LDL, chylomicron remnants and either apoB100 or B48, and are referred to as apoB-carrying lipoproteins.

### Preparation of ApoE-Enriched Lipoproteins

Mouse apoE-free (E^−^) lipoproteins were enriched with human apoEs as described by Clavey et al. [48]. Briefly, 20 µg/ml (protein) of apoE-free (E^−^) lipoproteins were incubated on a nutating shaker for 1 h at room temperature with apoE3 or E4 (concentrations indicated in figure legends). Total cholesterol and free cholesterol in apoE-free and apoE-enriched lipoproteins were measured by colorimetry (Wako Diagnostics; Richmond, VA) [49]. The esterified cholesterol was calculated by subtraction of FC from TC [49]. A 100 µl of apoE-free or apoE-enriched lipoproteins was fractioned using fast performance liquid chromatography (FPLC) (Akta FPLC900; Amersham Biosciences, Piscataway, NJ) equipped with a Superose 6 column (GE Life Sciences; Pittsburgh, PA) in a buffer containing 0.15 M NaCl, 10 mM Na_2_H P0_4_, 1 mM EDTA, and 0.02% NaN_3;_ pH 7.4, at a rate of 0.5 ml/min. Forty fractions (0.5 ml) were collected. Under these conditions, fractions 14–17 contained very low-density lipoprotein (VLDL), fractions 18–25 contained LDL and fractions 26–40 contained high density lipoprotein (HDL). Total cholesterol in each fraction was measured as described above.

### Western Blotting

RAW 264.7 cells were maintained in DMEM containing 10% fetal bovine serum (FBS), 100 U/ml penicillin and 100 µg/ml streptomycin at 37°C under 5% CO_2_ in 100-mm^2^ culture dishes; cultures were split upon reaching confluence. For western blot analysis, cells were grown to confluence in six-well plates, incubated in serum-free DMEM for 2.5 h and then treated with lipid-free or lipid-associated apoEs or E^−^ lipoproteins or culture medium alone for the time periods indicated in the figure legends. Cells were collected into 1.5 ml tubes and the medium removed following centrifugation at 1,000×g for 3 min. Cells were suspended in slightly hypotonic NET lysis buffer (100 mM NaCl, 1 mM sodium EDTA, 10 mM Tris, and 0.5% Triton X-100, pH 7.4) containing protease/phosphatase inhibitors (Sigma Aldrich) and lysed by three freeze/thaw (−80°C/room temperature) cycles, with gentle mixing. Supernatant protein fractions were isolated by centrifugation of the lysates at 16,000×g for 10 min. The supernatant containing 5–60 µg protein, based on the abundance of the studied proteins, was mixed with 2 × (v/v) urea loading buffer (8 M urea, 715 mM β-mercaptoethanol, 2% SDS, 125 mM Tris (pH 6.8), and 1% glycerol) and incubated at room temperature for 10 min before electrophoresis. The mixture was resolved on 6% SDS-PAGE gels made with 1 M Tris (pH 8.8) for separation of phosphorylated and non-phosphorylated Sp1, or on 10% SDS-PAGE made with 1.5 M Tris for separation of the other proteins studied. The proteins were then transferred to a polyvinylidene difluoride (PVDF) membrane. After blocking with 3% BSA, the PVDF membrane was incubated with solution containing phosphatase inhibitors (50 mM NaF, 0.1 mM sodium vanadate and 5 mM EDTA, pH 7.4) and primary antibodies against indicated proteins, and then with horseradish peroxidase-conjugated secondary antibodies. Immunoreactive bands were visualized using ECL-plus chemiluminescence reagent (GE Healthcare–Amersham) and analyzed with a GS-700 Imaging Densitometer (Bio-Rad, Hercules, CA).

### THP-1 Treatment

To convert THP-1 cells into macrophages, they were incubated with RPMI-1640 medium containing 10% FBS and 100 ng/ml of phorbol 12-myristate 13-acetate (PMA) for three days [50]. For treatment, the transformed cells were cultured with serum-free RPMI-1640 containing the same concentration of PMA for 2.5 h, and then treated with 3 µg/ml of apoE3 or apoE4, or culture medium alone for 5 h. Cell protein extracts were prepared for western blot analysis as discussed above.

### Quantitative Real-Time Reverse Transcription PCR

Confluent RAW264.7 cells in six-well plates were incubated in serum-free DMEM for 2.5 h, and then treated with lipid-free or lipid-associated apoEs, or E^−^ lipoproteins or culture medium alone for the time periods indicated in the figure legends. Total RNA was extracted using Trizol reagent (Invitrogen) and subjected to reverse transcription using a High Capacity cDNA reverse transcription kit (Applied Biosystems). The resulting cDNAs were subjected to quantitative real-time PCR with an iCycler system (Bio-Rad; Hercules, CA). The following specific primers were used for amplification: ABCA1 forward: 4′-GCTACCCACCCTACGAACAA-3, reverse: 5′-GGAGTTGGATAACGGAAGCA-3′; and GAPDH forward: 5′-GAGCCAAAAGGGTCATCATC-3′, reverse: 5′-TAAGCAGTTGGTGGTGCAGG-3′. The expression levels of ABCA1 mRNA were normalized relative to GAPDH mRNA**.**

### Lipid Extraction and Cholesterol Content

Confluent RAW264.7 cells in six-well plates were incubated at 37°C with 20 µg/ml of E^−^ lipoproteins for 87 h followed by culture medium alone or 3 µg/ml of lipid-free apoE3 or apoE4 for an additional 6 h. To ensure uniformity among samples, cells were lysed in NET lysis buffer by repeated freeze/thaw cycles [51]. The lipid solvent (3 times the volume of the lysis buffer, and containing chloroform, isopropranol and NP-40 at a ratio of 7∶11:0.1, v/v) was mixed with the lysate at room temperature for 10 min. The phases were separated by centrifugation at 16,000 × g for 10 min. The lower lipid layer was transferred to a new tube and evaporated under vacuum. The dried pellet was resuspended in ethanol: diethylether (3∶1, v/v). Free and total cholesterol were measured using cholesterol assay reagents [49]. Based on our studies with added free cholesterol and cholesterol ester standards, about 95% of the cholesterol is recovered in the lipid phase.

### Cholesterol Efflux Assay

RAW264.7 cells were grown to 60% confluence in 24-well plates, and then incubated for 24–48 h with 1 µCi/ml of 1,2-^3^H(N)]-cholesterol (∼ 450,000 dpm/µCi) in the presence of 20 µg/ml of E^-^ lipoprotein in DMEM supplemented with 10% FBS. Unbound lipoprotein was removed by incubating the cells at 4°C×15 min with 500 U/ml of heparin [52] dissolved in DMEM, followed by gentle washing of the cells with DMEM. For study of the effect of apoEs on apoAI-mediated cholesterol efflux, the radiolabeled cells were treated with 3 µg/ml of lipid-free apoE as indicated for 5 h at 37°C in DMEM. Surface bound apoE was removed with 500 U/ml of heparin. The cells were then washed gently with DMEM [52], [53]. Twenty µg/ml of apoAI or the same volume of vehicle was added to the cultures and incubated for additional 2 h at 37°C. The culture medium was collected, and cells were lysed with 0.5 M NaOH. The lysate and medium were each mixed with scintillation fluid to assay radioactivity using a Tri-Carb 2300TR Liquid Scintillation Analyzer (Perkin Elmer). Cholesterol efflux was expressed as the percentage of radioactivity in the medium compared to the total radioactivity (cells plus medium). The apoAI-mediated cholesterol efflux was calculated by subtracting the efflux in the absence of apoAI from the efflux in the presence of apoAI. For studying the effect of equimolar amount of apoEs and apoAI on cholesterol efflux, the radiolabeled macrophages were treated with 0.44 µM of apoAI (12.35 µg/ml), or apoE3 or apoE4 (15 µg/ml), or culture medium alone for 6 h. Cholesterol efflux was determined by measuring the radioactivities in the culture medium and cell lysates as described above.

### Statistics

Data are reported as the mean ± SEM. Statistical significance for the differences between treatment and control groups were analyzed by analysis of variance followed by Tukey’s post-hoc test or Student’s unpaired *t*-test. Statistix software (Statistix, Tallahassee, FL) was used for statistical analyses.

## Results

### Enrichment with ApoE Under Cell-Free Conditions does not Alter the Lipid Profile of ApoE-Free Lipoproteins

**Figure 1:**
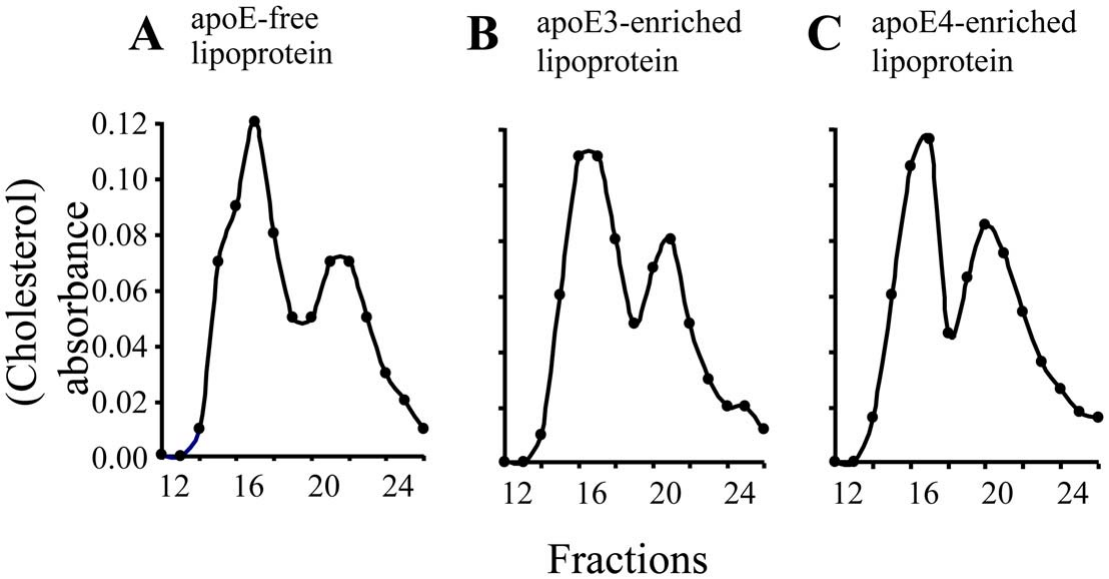
Effect of apoE enrichment on lipoprotein cholesterol profile. Fifty µg of apoE-free (E^−^) lipoproteins (<1.063) were incubated with 5 µg of apoE3 or apoE4 or an equivalent volume of vehicle at RT for 1 h. Cholesterol profiles in E^-^ lipoproteins (A), apoE3- (B), and apoE4-enriched (C) lipoproteins were analyzed with fast protein liquid chromatography.

The apoE-free (E^−^) mouse lipoproteins with density <1.063 g/ml contain VLDL and LDL particles. In order to compare the effect of lipid-associated apoE isoforms on gene expression and cholesterol efflux, human apoE3 or apoE4 was incorporated into E^−^ lipoproteins. As the FPLC traces in Fig. 1 show, enrichment of apoE under cell-free conditions did not significantly alter the cholesterol profile of the E^−^ lipoproteins. The ratio of cholesterol content in VLDL/LDL in E^−^ lipoproteins is 0.40/0.60. We previously reported that ∼70% of cholesterol was distributed in the VLDL fractions of *apoE^−/−^* mice, when mouse plasma samples were directly analyzed by FPLC [54]. These observations suggest that some large size lipoprotein particles are lost during ultracentrifugal isolation. Nevertheless, enrichment with apoEs did not alter cholesterol distribution in these lipoprotein particles, *i.e.*, ∼40 and ∼60% of cholesterol distributes, respectively, in the VLDL and LDL particles in apoE3- and apoE4-enriched lipoproteins. The data in Table 1 also show that enrichment with apoEs did not alter the level of cholesterol esters and triglycerides in E^−^ lipoproteins.

**Table 1:** Lipid content of fractionated lipoproteins.

Lipoprotein	Total Cholesterol (mg/dl)	Cholesterol Ester (mg/dl)
**E^−^**	4.871	0.379
**E^−^/E3**	4.878	0.378
**E^−^/E4**	4.871	0.377

ApoE-free (E^–^) lipoproteins were incubated on a nutating shaker for 1 h at room temperature with apoE3 (E^–^/E3) or E4 (E^–^/E4). The ratio of apoEs to E^–^ lipoprotein was 1 to 10 (protein to protein). The lipids were then fractionated by FPLC. Total cholesterol (TC) and free cholesterol (FC) in apoE-free and apoE-enriched lipoproteins were measured by colorimetry. Cholesterol ester was calculated as the difference between TC and FC.

### Inefficiency of ApoE4 at Inducing ABCA1 Expression in Mouse Macrophages

**Figure 2:**
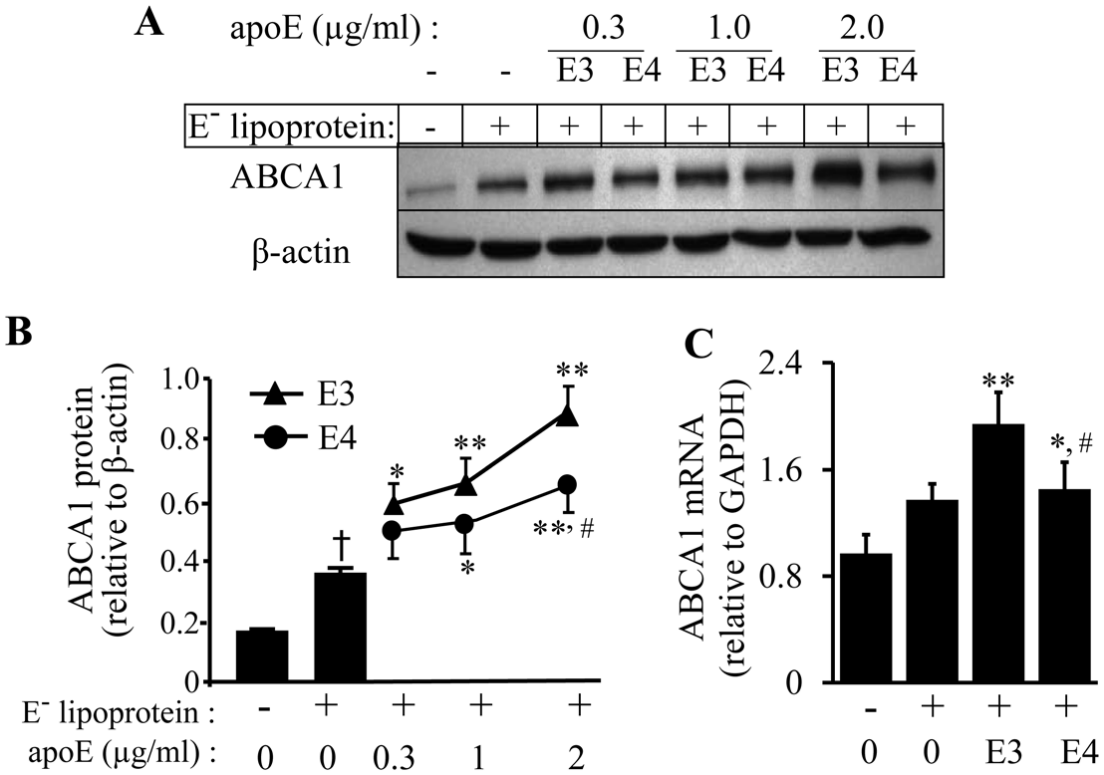
Concentration-dependent effect of lipoprotein-associated apoE isoforms on ABCA1 expression. (A–B) RAW 264.7 macrophages were incubated for 5 h at 37°C with 20 µg/ml of apoE-free (E^−^) lipoprotein, 20 µg/ml of E^−^ lipoproteins carrying the indicated concentrations of apoE3 (E3) or apoE4 (E4), or with culture medium alone as a control (Ctrl). ABCA1 protein levels were determined by immunoblotting, and normalized relative to β-actin. (C) RAW 264.7 macrophages were incubated for 5 h at 37°C with 20 µg/ml of apoE-free (E^-^) lipoprotein, 20 µg/ml of lipoproteins carrying 2 µg/ml of apoE3 (E3) or apoE4 (E4), or culture medium alone (Ctrl). ABCA1 mRNA levels were determined by quantitative real-time RT-PCR and normalized relative to GAPDH mRNA. Data represent the mean ± SEM from four separate experiments. ^†^ *P*<0.05 relative to untreated controls; *P<0.05 or **P<0.01 relative to cells treated with E^-^ lipoprotein; **^#^** *P*<0.05 relative to cells treated with apoE3-carrying lipoproteins.

Our previous studies demonstrated that apoB-carrying lipoproteins obtained from both wild-type and *apoE^−/−^* mice were able to induce ABCA1 expression and that the magnitude of this induction was greater using apoE-carrying lipoproteins than E^−^ lipoproteins [44], [45]. These findings suggested a role for lipid-associated apoE in the up-regulation of ABCA1. The present study compared the ability of apoE3 and E4 to induce ABCA1 expression. As shown in Fig. 2, E^−^ lipoproteins alone enhanced ABCA1 mRNA and protein expression. However, enrichment with either apoE3 or apoE4 augmented the ability of the lipoproteins to up-regulate ABCA1 protein (Figs. 2A, B). ABCA1 protein levels increased as a function of the amount of apoE incorporated into the lipoproteins. Specifically, the ABCA1 protein levels in cells treated with lipoproteins enriched with 0.3, 1 and 2 µg/ml of apoE3 were about 69, 89, and 150% higher, respectively, than in cells treated with E^−^ lipoproteins alone. The data in Fig. 2A–B also show that the amount of ABCA1 protein induced by apoE4-enriched lipoproteins was less than that induced by apoE3-enriched lipoproteins. Thus the ABCA1 protein levels in cells treated with lipoproteins enriched with the same doses of apoE4 were only 43, 54, and 86% higher, respectively, than cells treated with E^−^ lipoprotein alone. The data in Fig. 2C show that the ABCA1 mRNA levels in cells treated with 2 µg/ml of apoE4- and apoE3-enriched lipoproteins were about 6 and 42% higher, respectively, than in cells treated with E^−^ lipoproteins alone. The ABCA1 mRNA levels between cells treated with apoE-free and apoE4-enriched lipoproteins was not statistically significant. In contrast, the ABCA1 mRNA level was significantly higher in cells treated with apoE3-enriched lipoproteins than in cells treated with apoE-free lipoproteins.

**Figure 3:**
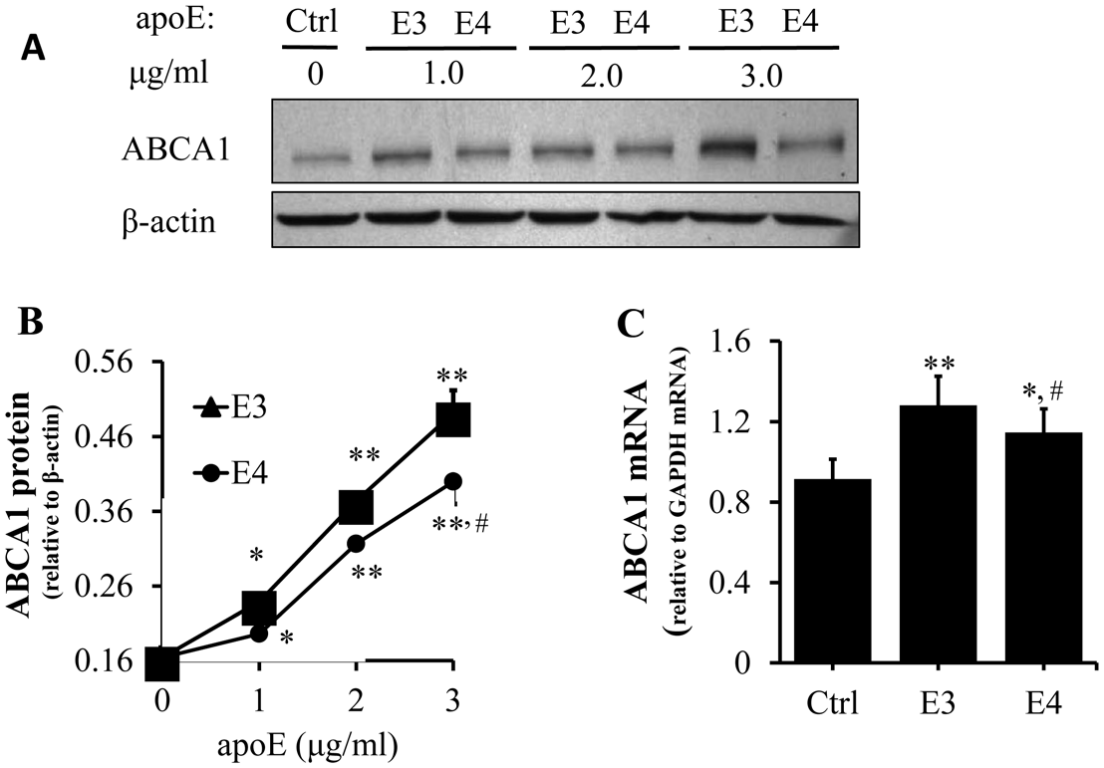
Concentration-dependent effect of lipid-free apoE isoforms on macrophage ABCA1 expression. (A–B) RAW 264.7 macrophages were incubated for 5 h at 37°C with the indicated concentrations of apoE3 (E3) or apoE4 (E4) or with culture medium alone as a control (Ctrl). ABCA1 protein levels were determined by immunoblotting. (C) RAW 264.7 macrophages were incubated for 5 h at 37°C with 3 µg/ml of apoE3 (E3) or apoE4 (E4), or with culture medium alone as a control (Ctrl). ABCA1 mRNA levels were determined by quantitative real-time RT-PCR, normalized relative to GAPDH mRNA. Data represent the mean ± SEM from 3–4 separate experiments. *P<0.05 or **P<0.01 relative to untreated controls; **^#^** *P*<0.05 relative to apoE3.

To determine whether the deficiency of apoE4 in inducing ABCA1 expression was related to being associated with a lipid complex, we treated the cells with lipid-free apoE isoforms. As the data in Figs. 3A, B show, both apoE3 and apoE4 induced ABCA1 protein expression, independent of lipid association, in a dose-dependent manner, although the magnitude of the increase induced by apoE4 was again significantly less than that induced by apoE3. Further ABCA1 mRNA levels in cells treated with apoE4 and apoE3 were ∼26 and 40% higher, respectively, than in untreated control cells (Fig. 3C). Similarly, lipid-free apoE4 induced less ABCA1 mRNA than the same concentration of apoE3. These results indicate that lipid-free apoE4 is less effective than apoE3 at inducing ABCA1 at both the mRNA and protein levels. These findings, together with the data in Fig. 2, suggest that apoE4, regardless of its lipid-free or lipid-associated form, manifests a reduced potential to induce ABCA1 expression as compared to apoE3.

### Inefficiency of ApoE4 at Inducing Cholesterol Efflux and Removing Accumulated Cholesterol in Mouse Macrophages

**Figure 4:**
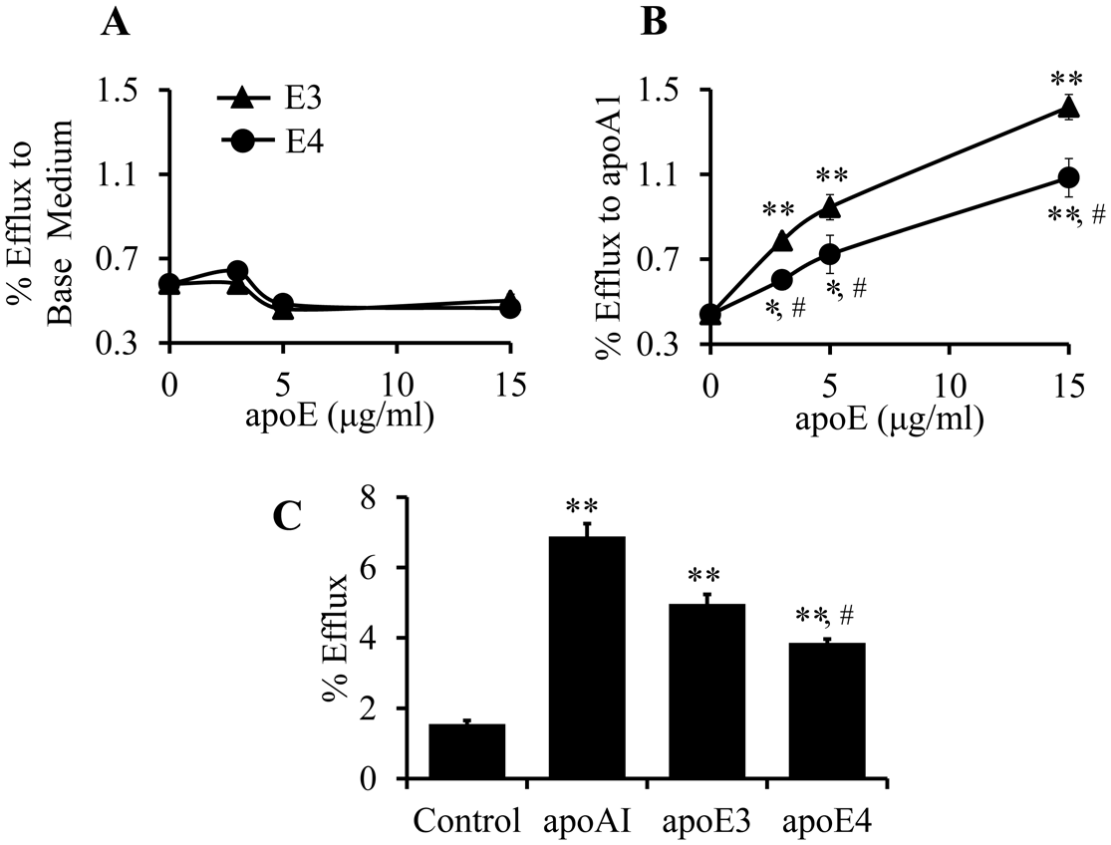
Effect of lipid-free apoE and apoAI on cholesterol efflux. (A–B) RAW 264.7 macrophages were incubated at 37°C for 48 h with 1 µCi/ml of ^3^H-cholesterol and 20 µg/ml apoE-free (E^−^) lipoprotein, followed by incubation with the indicated amount of apoE3 (E3) or apoE4 (E4) for an additional 5 h. Thereafter, cells were incubated with 20 µg/ml of apoAI or base medium alone for 2 h. Cholesterol efflux was determined as described in the Materials and Methods. ApoAI mediated cholesterol efflux was calculated as the total efflux in the presence of apoAI minus the efflux to the base medium without apoAI. (C) The cells were loaded with ^3^H-cholesterol as abovementioned, and then incubated with 0.44 µM of lipid free apoA1 (12.35 µg/ml), apoE3 or apoE4 (15 µg/ml), or culture medium alone as a control (ctrl) for 6 h. Total cholesterol efflux was determined as described in the Materials and Methods. Data represent the mean ± SEM from 3–4 separate experiments. *P<0.05 or **P<0.01 relative to controls; **^#^** *P*<0.05 relative to apoE4.

An important function of ABCA1 is to transport cholesterol to apoAI [55]. To determine whether the poor induction of ABCA1 expression by apoE4 is associated with a reduced apoAI-mediated cholesterol efflux, lipid-laden cells were pretreated with lipid-free apoEs for 5 h to stimulate ABCA1 expression, and then incubated with or without apoAI for 2 h, followed by measurement of cholesterol efflux. The data in Fig. 4A show that apoE pretreatment did not significantly affect apoAI-independent cholesterol efflux. Specifically, the level of radiolabeled cellular cholesterol efflux in cells pretreated with apoE3 or apoE4, or in the untreated control cells was comparable in the absence of apoAI. However, pretreatment of macrophages with apoE3 or apoE4 increased apoAI-mediated cholesterol efflux in a dose-dependent manner, and cholesterol efflux in cell pretreated with apoE3 was significantly higher than in cells treated with the same doses of apoE4 (Fig. 4B). Specifically, the apoAI-mediated cholesterol efflux in macrophages pretreated with 3, 5 and 15 µg/ml of apoE3 was 79, 115 and 223% higher, respectively, than that in the control cells without apoE pretreatment, while the cholesterol efflux level in cells pretreated with the same doses of apoE4 was 37, 64 and 147% higher, respectively, than in the control cells (Fig. 4B). These findings suggest that the relatively low induction of ABCA1 expression by apoE4 was also associated with a reduced apoAI-mediated cholesterol efflux in macrophages.

Like apoAI, apoEs can also function as an acceptor in ABCA1-mediated cholesterol efflux [24], [56]. The data in Fig. 4C show the effect of equimolar amount of apoEs and apoAI on cholesterol efflux. In this study, lipid-laden cells were treated with 0. 44 µM of apoAI or apoEs, directly followed by measurement of cholesterol efflux. Similar to the data shown in Fig. 4B, cholesterol efflux was significantly lower in macrophages treated with apoE4 than apoE3 (Fig. 4C). In addition, cholesterol efflux was significantly higher in cells treated with apoAI than in cells treated with apoE3 or apoE4.

**Figure 5:**
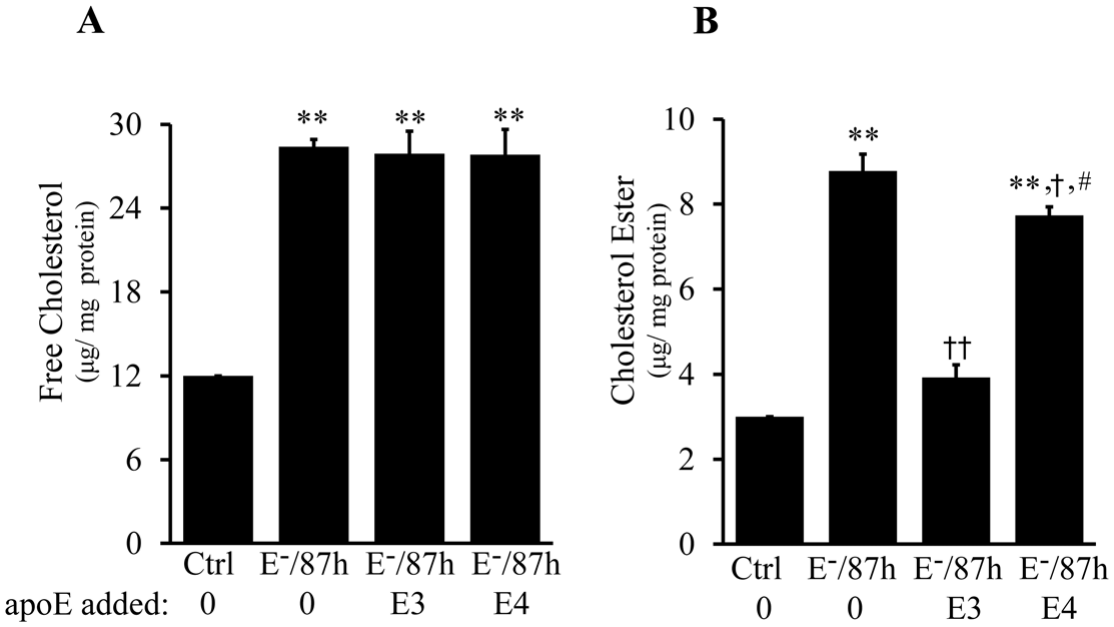
Effect of lipid-free apoE isoforms on the cholesterol level of lipid-laden macrophages. RAW264.7 macrophages were incubated at 37°C for 87 h with 20 µg/ml of apoE-free (E^−^) lipoproteins, followed by an additional 6h incubation with culture medium alone (E^−^/87 h), 3 µg/ml of lipid-free apoE3 (E^−^/87 h + E3), or apoE4 (E^−^/87 h + E4). Cells without lipoprotein and apoE treatments were used as controls (Ctrl). (A–B) Cellular total and free cholesterols were measured colorimetrically and esterified cholesterol was calculated from the difference between total and free cholesterol. Data represent the mean ± SEM from three independent experiments. **P<0.01 relative to control; ^†^ *P*<0.05 or^ ††^ *P*<0.01 relative to E^−^/87 h; **^#^** *P*<0.05 relative to E^−^/87 h + E3.

Cholesterol efflux is a mechanism by which excess cholesterol is removed from the cell. Having demonstrated the impaired ability of apoE4 to promote cholesterol efflux through ABCA1, we then compared the direct effects of apoE4 with apoE3 on removal of cholesterol from chronically lipid-laden macrophages. As can be seen in Figs. 5A, B, incubation of macrophages with 20 µg/ml E^−^ lipoproteins for 87 h elevated both esterified and free cholesterol by more than 2 fold as compared to control cells not treated with E^−^ lipoproteins. Accumulation of cholesterol esters transforms macrophages into foam cells. The data in Fig. 5B show that treatment of the lipid-laden cells with 3 µg/ml lipid-free apoE3 for 6 h reduced the macrophage cholesterol ester levels by 55%, as compared to cells treated with E^−^ lipoproteins alone. It is important to note that the cholesterol removal in cells treated with apoE3 (Fig. 5) was faster than cholesterol efflux (Fig. 4C). For example, the cholesterol removal in cells treated with 3 µg/ml of apoE3 for 6 h (Fig. 5) and cholesterol efflux in cells treated with 15 µg/ml of apoE3 for the same time period (Fig. 4) were about 14% and 6.5%, respectively. It is known that the effectiveness of cholesterol efflux is largely dependent on the levels of cellular cholesterol and ABCA1 protein [57]. The higher the cellular cholesterol and ABCA1 levels, the faster the cholesterol efflux. However, the data in Fig. 4 were obtained from cells incubated with E^−^ lipoproteins and ^3^H cholesterol for 48 h, while the data in Fig. 4 were obtained from cells incubated with E^−^ lipoproteins for 87 h. The accumulated cholesterol level is therefore higher in cells treated with 20 µg/ml E^−^ lipoproteins for 87 h than for 48 h (∼37 vs. ∼29 µg/mg protein).

The data in Fig. 5 also show that the cholesterol removal induced by the same dose of apoE4 was significantly lower than apoE3, i.e., treatment of the cells with 3 µg/ml of apoE4 for 6 h reduced the cholesterol ester by only ∼12% (Fig. 5B). This is consistent with the findings of others that lipid-free apoE3 is more effective than apoE4 at removing cholesterol from cells [36], [37]. Neither apoE3 nor apoE4 significantly altered the free cholesterol level in the lipid-laden cells for the duration of treatment (Fig. 5A).

### Inefficiency of ApoE4 at Inducing Sequential PI3K, PKCζ, and Sp1 Phosphorylations in Mouse Macrophages

**Figure 6:**
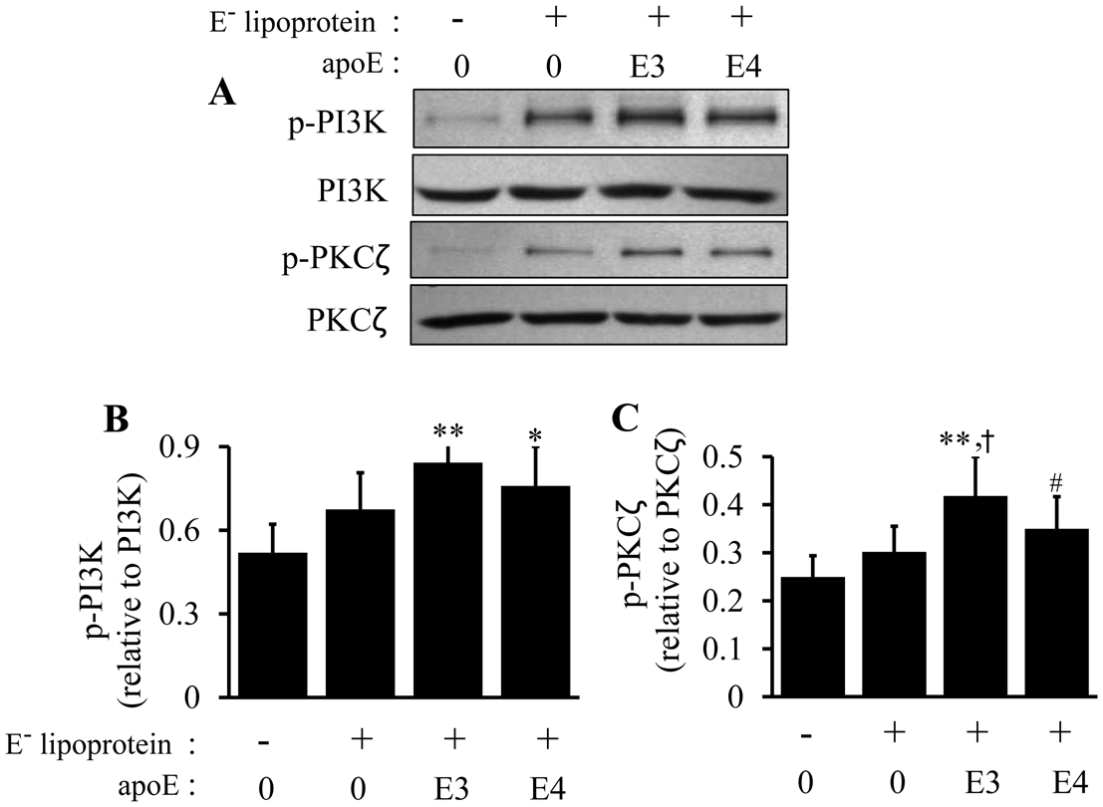
Effect of lipoprotein-associated apoE isoforms on PI3K and PKCζ phosphorylation. RAW 264.7 macrophages were incubated at 37°C for 5 h with 20 µg/ml of apoE-free (E^−^) lipoprotein, 20 µg/ml of E^−^ lipoproteins containing 2 µg/ml of apoE3 (E3) or apoE4 (E4) or culture medium alone as a control (Ctrl). (A–C) Phosphorylated PI3K and PKCζ were determined by immunoblotting. Data represent the mean ± SEM from 3–4 separate experiments. *P<0.05 or **P<0.01 relative to controls; ^†^ *P*<0.05 relative to E^−^; **^#^** *P*<0.05 relative to apoE3.

Our laboratory previously demonstrated that phosphorylation of PI3K, PKCζ and Sp1, in this sequence, is a mechanism by which apoB-carrying lipoproteins induce ABCA1 expression in RAW 264.7 macrophages [45]. The present report compared the effects of apoE4 versus apoE3 on the induction of phosphorylated PI3K (p-PI3K), phosphorylated PKCζ (p-PKCζ) and phosphorylated Sp1 (p-Sp1) in macrophages. As can be seen in Fig. 6, both lipid-associated apoE3 and apoE4 increased PI3K and PKCζ phosphorylations that were above those induced by E^−^ lipoproteins alone, *i.e.,* the same dose (20 µg/ml) of apoE-free, and apoE3- or apoE4-enriched lipoproteins elevated p-PI3K level by 29, 62 and 45%, respectively, in macrophages. Likewise, p-PKCζ increased by 20, 67 and 40% in cells treated with apoE-free, and apoE3- or apoE4-enriched lipoproteins, respectively.

**Figure 7:**
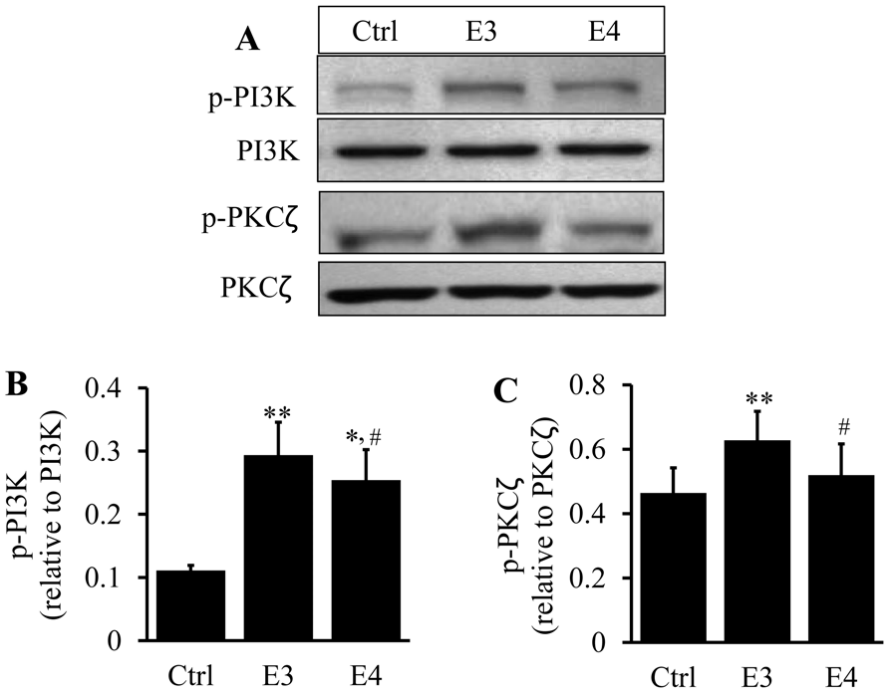
Effect of lipid-free apoE isoforms on PI3K and PKCζ phosphorylations. RAW 264.7 macrophages were incubated at 37°C for 5 h with 3 µg/ml of lipid-free apoE3 (E3) or apoE4 (E4) or culture medium alone as a control (Ctrl). (A–C) Phosphorylated PI3K and PKCζ were determined by immunoblotting. Data represent the mean ± SEM from 4 separate experiments. *P<0.05 or **P<0.01 relative to controls; **^#^** *P*<0.05 relative to apoE3.

In the lipid-free state, apoE3 also exhibited greater effectiveness at stimulating PI3K and PKCζ phosphorylations than apoE4. Specifically, the level of phosphorylated PI3K and phosphorylated PKCζ was ∼160 and130% higher in cells treated with apoE3 and apoE4 than in untreated control cells, respectively (Fig. 7). Likewise, p-PKCζ levels were significantly greater in cells treated with apoE3 than in cells treated with apoE4. These data indicate that the ability of apoE4 to induce PI3K and PKCζ phosphorylation were reduced compared to the same concentration of apoE3, the so called normal isoform of human apoE.

**Figure 8:**
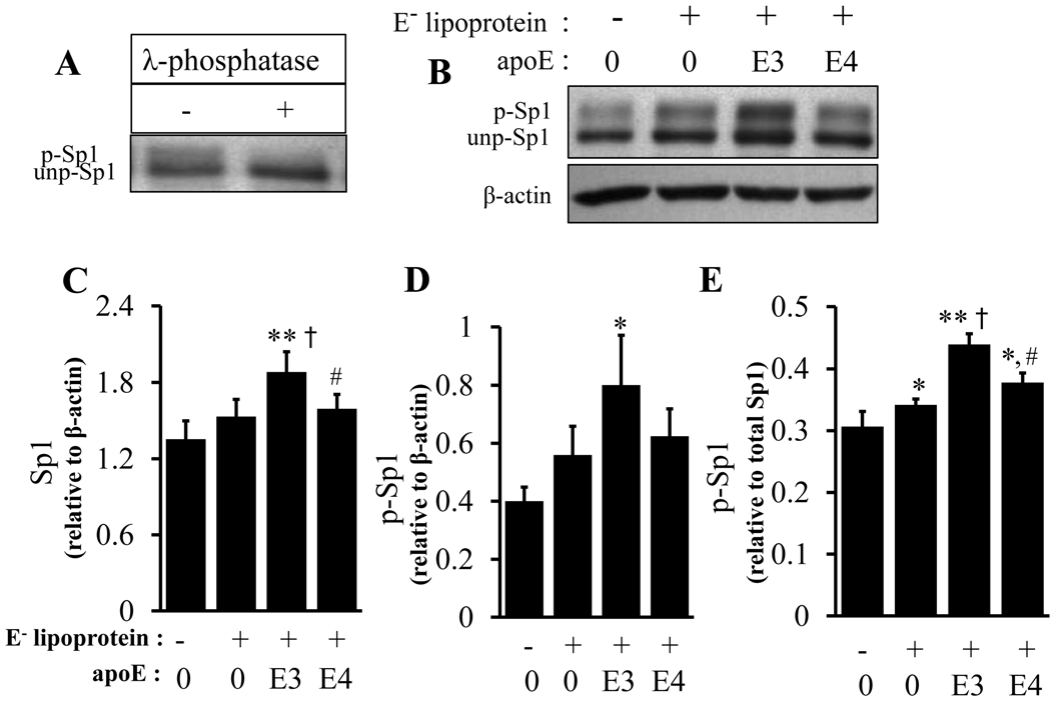
Effect of lipid-associated apoE isoforms on Sp1 expression and phosphorylation. (A) RAW 264.7 cell lysates were incubated for 5 h with or without λ-phosphatase and Sp1 levels determined by immunoblotting. The upper band (p-Sp1) was determined to be the phosphorylated form (p-Sp1) and the lower band (unp-Sp1), the unphosphorylated form. (B–E) RAW 264.7 macrophages were incubated at 37°C for 5 h with 20 µg/ml of apoE-free (E^−^) lipoprotein, 20 µg/ml of E^−^ lipoproteins containing 2 µg/ml of apoE3 (E3) or apoE4 (E4) or culture medium alone (Ctrl). The level of phosphorylated Sp1 (top band) was expressed relative to the total Sp1 protein level (the sum of the top and bottom bands relative to actin) or actin. Data represent the mean ± SEM from 3–4 separate experiments. *P<0.05 or **P<0.01 relative to controls; ^†^ *P*<0.05 relative to E^−^; **^#^** *P*<0.05 relative to apoE3.

**Figure 9:**
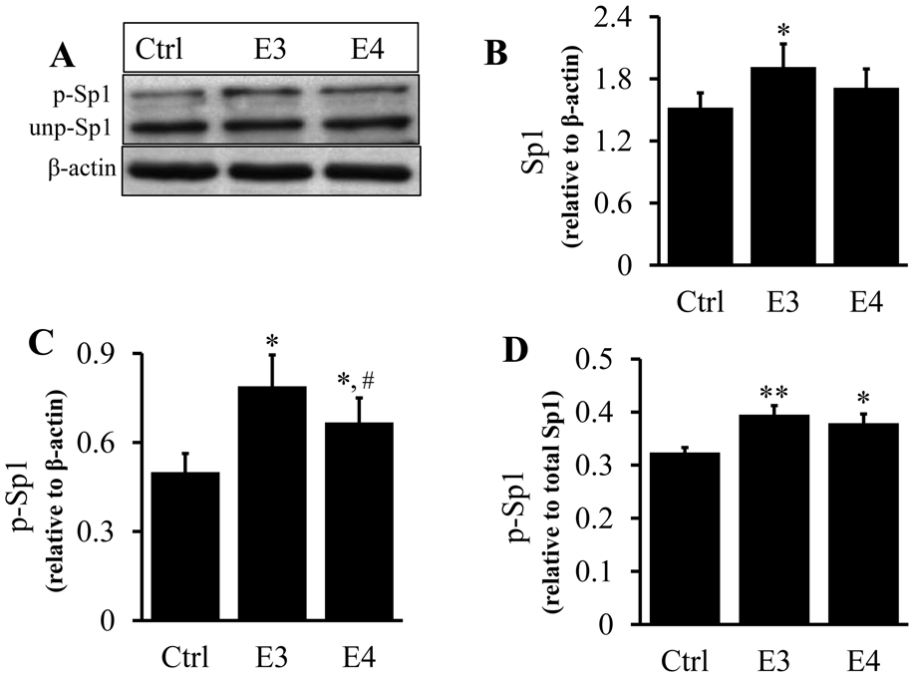
Effect of lipid-free apoE isoforms on Sp1 expression and phosphorylation. (A–D) RAW 264.7 macrophages were incubated at 37°C for 5 h with 3 µg/ml of lipid-free apoE3 (E3) or apoE4 (E4) or culture medium alone as a control (Ctrl). Sp1 protein was determined by immunoblotting. The level of phosphorylated Sp1 (top band) was expressed relative to the total Sp1 protein level (the sum of the top and bottom bands relative to actin) or actin. Data represent the mean ± SEM from 3–4 separate experiments. *P<0.05 or **P<0.01 relative to controls; **^#^**P<0.05 relative to apoE3.

As the representative images show, two Sp1 immunoreactive bands were detected on western blots (Figs. 8A- B, 9A). To determine whether the slower migrating form of Sp1 (top band) was generated by phosphorylation, we incubated cell lysates with λ-phosphatase before western blot analysis. Fig. 8A shows that λ-phosphatase abolished the top band, suggesting that the slower migrating form of Sp1 was a result of Sp1 phosphorylation. We thus determined the phosphorylation of Sp1 using the ratio of the intensity of the top band versus the sum of the top and bottom immunoreactive bands.

Data in Figs. 8 B–D, 9A–C show that treatment of macrophages with apoE-free, apoE3- or apoE4-enriched lipoproteins, as well as lipid-free apoE, elevated both phosphorylated and total Sp1 proteins. However, the phosphorylated Sp1 level increased to a greater extent, significantly elevating the ratio of phosphorylated versus total Sp1 proteins in cells treated with lipid-free apoE or lipoproteins, when compared to the untreated controls. In detail, 20 µg/ml of apoE-free, apoE3- or apoE4-enriched lipoproteins increased the total Sp1 level by 13, 40, and 18%, respectively, relative to untreated controls, while the same dose of these lipoproteins increased p-Sp1 by 40, 100, and 56%, respectively (Fig. 8D). Correspondingly, the ratio of p-Sp1 relative to total Sp1 was increased by 15, 46, and 32% in apoE-free, apoE3- or apoE4-enriched lipoproteins, respectively, compared to untreated controls (Fig. 8E). The Sp1 phosphorylation induced by apoE3-enriched lipoprotein was significantly higher than that induced by apoE-free and apoE4-enriched lipoproteins (Fig. 8E). In the lipid-free form, apoE3 and apoE4 elevated the ratio of phosphorylated Sp1 versus β-actin by 65and 33%, respectively, compared to untreated control cells, while the level of phosphorylated Sp1 was significantly lower in apoE4-treated macrophages than in apoE3-treated cells (Fig. 9C). The ratio of p-Sp1 relative to total Sp1 was slightly smaller in apoE4-treated macrophages than in apoE3-treated cells (23 versus 18%), though the difference was not statistically significant (Fig. 9D).

### Inefficiency of ApoE4 at Inducing ABCA1 Expression and Promoting PKCζ and Sp1 Phosphorylations in Human THP-1 Macrophages

**Figure 10:**
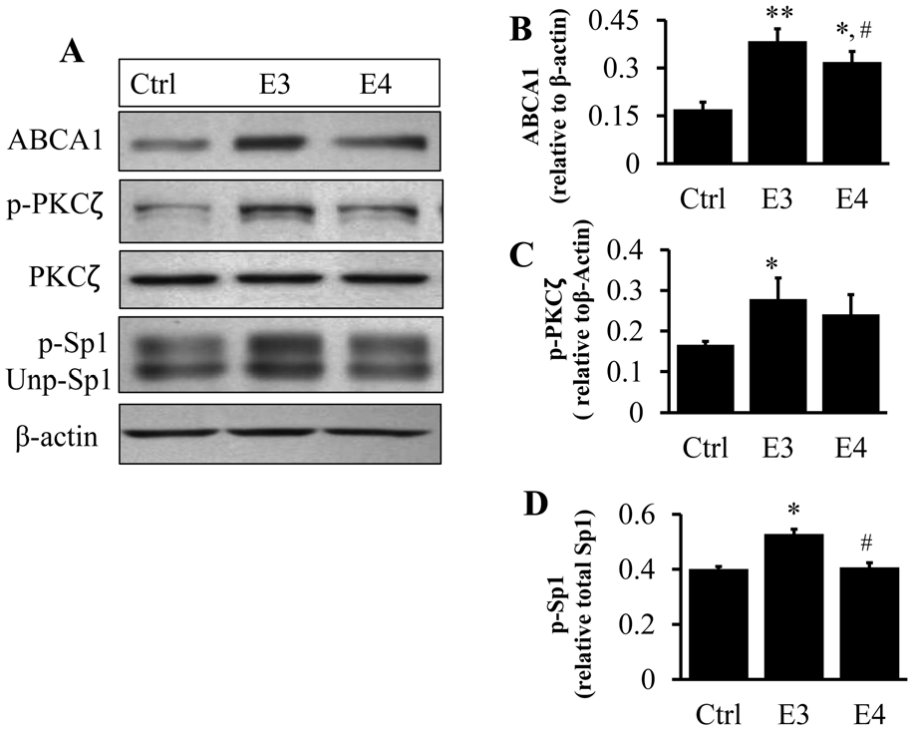
Effect of lipid-free apoE isoforms on phosphorylation of PKCζ and Sp1, and ABCA1 expression in human THP-1 macrophages. (A–D) Human macrophage THP-1 cells were treated with 3 µg/ml of lipid-free apoEs for as described under Materials and Methods and the protein levels of ABCA1, p-PKCζ and Sp1 determined by immunoblotting. *P<0.05 or **P<0.01 relative to controls; ^#^ *P*<0.05 relative to apoE3.

To verify if the human THP-1 macrophage cell line was also able to respond to lipid-free apoEs like mouse macrophages, we treated THP-1 cells with 3 µg/ml of lipid-free apoE3 or apoE4. The data in Fig. 10 show that, like RAW 264.7 mouse macrophages, apoE4 was deficient at inducing ABCA1 expression compared to apoE3 using human macrophages. In addition, the propensity of apoE4 to induce PKCζ and Sp1 phosphorylations was reduced relative to apoE3. These findings suggest that the PKCζ-Sp1-ABCA1 signaling pathway is not limited to RAW 264.7 macrophages, and may represent a general cascade mediated by human apoE isoforms.

## Discussion

Data from the present report, for the first time, demonstrated an additional deficiency of apoE4 besides its inefficiency at recycling. Specifically, we observed that apoE4, using either a lipid-free or lipoprotein-associated form, was deficient in induction of ABCA1 expression in macrophages as compared to apoE3. In addition, apoE4 showed a reduced ability to induce apoAI-mediated cholesterol efflux and to remove cholesterol from lipid-laden cells. ABCA1 is a membrane protein that exports excess cholesterol derived from internalized lipoproteins to apoAI, initiating nascent HDL formation. Mutation of ABCA1 induces intracellular cholesterol accumulation and increases susceptibility to atherosclerosis in humans [58] and in animal models [59]. Thus, a deficiency in the ability of apoE4 to induce macrophage ABCA1 expression and apoAI-mediated cholesterol efflux might contribute to the pathogenesis of atherosclerosis. Indeed, ε4 carriers have a higher susceptibility to coronary heart diseases than ε3 carriers [31]-[33].

Another novel finding from the present report is that the capability of apoE4 to induce Sp1 phosphorylation was reduced as compared to apoE3. Sp1 is one of the transcription factors that controls ABCA1 transcription [45]. We previously demonstrated that activation of Sp1 is a mechanism by which mouse apoB-carrying lipoproteins and lipid-free human apoE3 induce ABCA1 expression [45], [46]. Specifically, we observed that lipid-free apoE3 and apoB-carrying lipoproteins increased Sp1 phosphorylation and enhanced Sp1 binding to the ABCA1 promoter region. Inhibition of Sp1 DNA binding reduced ABCA1 expression, while mutation of the Sp1 binding motif in the ABCA1 promoter suppressed ABCA1 promoter activity induced by apoE3 and apoB-carrying lipoproteins. The impaired induction of Sp1 phosphorylation thus might be a mechanism for the reduced efficacy of apoE4 compared to apoE3 to up-regulate ABCA1 expression.

We previously demonstrated that activation of PKCζ is responsible for both Sp1 phosphorylation and the elevated ABCA1 expression induced by apoE/apoB-carrying lipoproteins [45] and lipid-free apoE3 [46]. Specifically, we observed that treatment of macrophages with mouse apoE/apoB-carrying lipoproteins increased PKCζ phosphorylation and induced physical interaction of PKCζ and Sp1. In addition, transfection of macrophages with a kinase dead PKCζ or treatment of the cells with a PKC inhibitor attenuated Sp1 phosphorylation and ABCA1 promoter activity induced by apoE/apoB-carrying lipoproteins and lipid-free apoE3. Moreover, we observed that the PKCζ phosphorylation induced by apoE/apoB-carrying lipoproteins and lipid-free apoE3 was reduced by inhibition of PI3K. These data suggest that the activation of the PI3K-PKCζ cascade is critical for Sp1 phosphorylation and ABCA1 expression. In the present report, we observed that apoE4 showed a reduced ability to induce PI3K and PKCζ phosphorylation. Taken together, the deficiency in activation of the PI3K-PKCζ-Sp1 cascade is likely responsible for the reduced induction of ABCA1 expression by apoE4.

It has been known that apoE triggers signaling transduction by interaction with membrane receptors [60]. A recent study from our laboratory showed that activation of VLDL receptor (VLDLR) and apoE receptor-2 (apoER2) is at least partially responsible for the induction of ABCA1 by lipid-free apoE3 [46]. Specifically, we observed that reelin, a specific ligand for VLDLR and apoER2 is able to mimic the up-regulatory effect apoE3 on ABCA1 expression. The increased ABCA1 expression by both apoE3 and reelin is associated with increased phosphorylation of disabled-1 (Dab1), PI3K, PKCζ and Sp1. Moreover, reelin- or apoE3-mediated up-regulation of ABCA1 expression, and phosphorylation of Dab1, PI3K, PKCζ and Sp1 were suppressed by knockdown of VLDLR or apoER2 with small interfering RNAs. It appears that stimulation of VLDLR and apoER2 by reelin or apoE activates the Dab1, PI3K-PKCζ-Sp1 signaling cascade, which in turn up-regulates ABCA1 expression. Though the interaction of apoE4 with VLDLR and apoER2 has not been studied in detail, indirect evidence supports the view that apoE4 is deficient in the VLDLR- and/or apoER2-mediated signaling transduction process. For example, ε4 allele carriers have an increased prevalence of Alzheimer’s disease, and a deficiency in signaling transduction involving VLDLR/apoER2-Dab1 has been suggested as a mechanism involved in Alzheimer’s disease. Taken together, it is likely that the reduced ability of apoE4 to induce ABCA1 expression and activate the PI3K-PKCζ-Sp1 cascade were initiated at the receptor level. However, further studies are needed to determine the differences between apoE3 and apoE4 in their interactions with membrane receptors, *e.g.*, VLDLR and apoER2, in order to provide direct evidence for this postulate.

In summary, apoE4 showed a reduced ability to induce macrophage ABCA1 expression as compared to the same concentration of apoE3. ABCA1 is a membrane protein that transports cellular cholesterol to apoAI. Correspondingly, apoE4 showed a reduced apoAI-mediated cholesterol efflux and a reduced ability to remove cholesterol from lipid-laden cells. A reduction in the removal of excess cholesterol could result in cellular cholesterol accumulation, and thus the deficiency of apoE4 in induction of ABCA1 expression and the consequent reduction in cholesterol efflux might contribute to the increased prevalence of atherosclerotic cardiovascular diseases in apoE4 allele carriers. In addition, data from the present report show that the phosphorylated level of PI3K, PKCζ and Sp1 induced by apoE4 was significantly less than that induced by apoE3. These findings suggest that apoE4 is deficient in the induction of this signaling pathway, which in turn reduces the induction of ABCA1 expression.
